# Dynamic segmental kinematics of the lumbar spine during diagnostic movements

**DOI:** 10.3389/fbioe.2023.1209472

**Published:** 2023-09-28

**Authors:** Paul McMullin, Darian Emmett, Andrew Gibbons, Kelly Clingo, Preston Higbee, Andrew Sykes, David T. Fullwood, Ulrike H. Mitchell, Anton E. Bowden

**Affiliations:** ^1^ Department of Mechanical Engineering, Brigham Young University, Provo, UT, United States; ^2^ Department of Exercise Sciences, Brigham Young University, Provo, UT, United States

**Keywords:** lumbar spine, segmental kinematics, dynamic, optical motion tracking, movement patterns

## Abstract

**Background:** *In vivo* measurements of segmental-level kinematics are a promising avenue for better understanding the relationship between pain and its underlying, multi-factorial basis. To date, the bulk of the reported segmental-level motion has been restricted to single plane motions.

**Methods:** The present work implemented a novel marker set used with an optical motion capture system to non-invasively measure dynamic, 3D *in vivo* segmental kinematics of the lower spine in a laboratory setting. Lumbar spinal kinematics were measured for 28 subjects during 17 diagnostic movements.

**Results:** Overall regional range of motion data and lumbar angular velocity measurement were consistent with previously published studies. Key findings from the work included measurement of differences in ascending *versus* descending segmental velocities during functional movements and observations of motion coupling paradigms in the lumbar spinal segments.

**Conclusion:** The work contributes to the task of establishing a baseline of segmental lumbar movement patterns in an asymptomatic cohort, which serves as a necessary pre-requisite for identifying pathological and symptomatic deviations from the baseline.

## 1 Introduction

Estimates of the prevalence of non-specific chronic low back pain (cLBP) state that anywhere from 20% to 90% of cLBP cases lack a known mechanical etiology, with most citations stating between 85% and 90% (White and Gordon, 1982; Koes, 2007; Maher et al., 2017). The widespread adoption of the biopsychosocial model of cLBP has supported non-mechanical diagnostic and treatment advancements, but even the best results of psychotherapies show effectiveness in only up to 50% of subjects with nonspecific cLBP (Ashar et al., 2022). With cLBP being the leading cause of disability worldwide and with an annual estimated $100 billion financial burden in the United States alone, it is vital to better understand the causes of cLBP. The widespread adoption of the biopsychosocial paradigm has helped many patients to find relief from their pain, however, more detailed knowledge of lumbar spinal mechanics, specifically spinal kinematics at the segmental level, may provide further knowledge to identify treatable movement phenotypes of cLBP (Cholewicki et al., 2019; Adam Quirk et al., 2022).

Data regarding spinal end range of motion (ROM) are widely available, however work over the last two decades has indicated that the entire kinematic behavior of the spine should be considered in diagnosing causes of cLBP. Intermediate motion, as well as rotational velocities, centers of rotation, and even rotational accelerations of the spine at both the overall and segmental levels have been suggested as promising avenues of understanding (Marras and Wongsam, 1986; Marras et al., 1993; Marras et al., 1995a; Zirbel et al., 2014; Pourahmadi et al., 2020). Comprehensive segmental kinematic understanding may provide a more sensitive method of phenotyping movement disorders, promoting more personalized treatment paradigms. Previous work indicates motion discrepancies between both subgroups of cLBP patients as well as between cLBP patients and pain-free individuals (Neblett et al., 2013; Hung et al., 2014; Laird et al., 2018; Moissenet et al., 2021). We anticipate that further refinement in movement phenotyping measurements would allow for more precise diagnostics as well as an insightful tool for monitoring patient recovery and responsiveness to treatment plans (Cholewicki et al., 2019; Adam Quirk et al., 2022).

There are few data available regarding dynamic *in vivo* segmental kinematics of the lumbar spine during activities of daily living. Previously published efforts have been small cohort studies of subjects using dual video fluoroscopy (Ahmadi et al., 2009; Bai et al., 2011; Passias et al., 2011; Xu et al., 2018) and optical motion tracking based on movement of percutaneous bone pins (Rozumalski et al., 2008; MacWilliams et al., 2013; MacWilliams et al., 2014). The principal challenges for implementing these technologies more broadly are cost, invasiveness of the measurement procedure, and in the case of biplane fluoroscopy, radiation exposure (Chleboun et al., 2012; Goodsitt et al., 2017). Thus, development of an objective standard for dynamic segmental spinal kinematics remains elusive, though researchers have called for further *in vivo* measurements of dynamic segmental kinematics of the spine (McGregor et al., 2001; Jones and Wilcox, 2008; Oxland, 2016).

The gold standard for dynamic kinematic measurement of human motion is based on skin-mounted marker tracking, and extensive previously published work has used marker tracking to measure overall motion of both the spine and spinal regions (e.g., overall lumbar, thoracic, and cervical kinematics). A number of studies have also developed methods of measuring *in vivo* segmental kinematics using motion capture systems (Ma et al., 2008; Rozumalski et al., 2008; MacWilliams et al., 2013; MacWilliams et al., 2014; Beaudette et al., 2017; Wang et al., 2021). Out of these, Beaudette et al. (2017) and Ma et al. (2008) report dynamic data in the sagittal plane. Additionally, Wang et al. (2021) reported end ROM measurements flexion/extension and for lateral bending.

To date, measurement of multi-planar dynamic segmental spinal motion has not been reported, likely due to challenges in obtaining sufficient resolution of markers on the skin, as well as anticipated challenges regarding soft tissue artifacts (STAs). All skin-mounted marker tracking solutions introduce the potential for STAs that can impede accurate kinematic measurements (Benoit et al., 2006; Andersen et al., 2012; Blache et al., 2017). Research quantifying STAs in spinal kinematic measurements indicate that optical motion tracking can provide estimates of lumbo-thoracic spinal kinematics in the sagittal plane (Zemp et al., 2014; Severijns et al., 2021), even in obese individuals (Menegoni et al., 2008). Quantification of STA in the cervical spine also indicates that reasonable estimates can be made in the sagittal and frontal planes (Wang et al., 2016). The novelty of the present work stems from the use of a high-density marker set to capture dynamic, multiplanar segmental lumbar spine kinematics (in contrast to previous studies reporting dynamic uniplanar motion or static end ROM utilizing other motion tracking methodologies) during a set of functional movements in a laboratory setting. The objective for the present work is the characterization of baseline kinematic features within a cohort of asymptomatic individuals as a reference point for novel wearable technologies which are currently in development (Baker et al., 2023), and as a prelude to comparisons with chronic low back pain individuals. Furthermore, the sharing of the data with other researchers via an open access data repository adds significant value to the study.

## 2 Materials and methods

### 2.1 Subjects

The study tested 28 subjects with no history of pathological back pain, including 13 females and 15 males between the ages of 20 and 59 years (see Table 1). Subjects were recruited from the community. Subject exclusion criteria included any history of cLBP, any occurrences of low back pain in the last month, and an inability to complete basic functional movements without pain. All subjects provided informed consent and the study protocol was approved by our institutional review board.

**TABLE 1:** Subject demographic information.

Subject demographic information	
Metric	Mean (standard deviation)
Age in *yrs*	25.3 (9.2)
Weight in *kg*	73.6 (14.2)
Height in *cm*	176.6 (8.3)

### 2.2 Data collection

**FIGURE 1:**
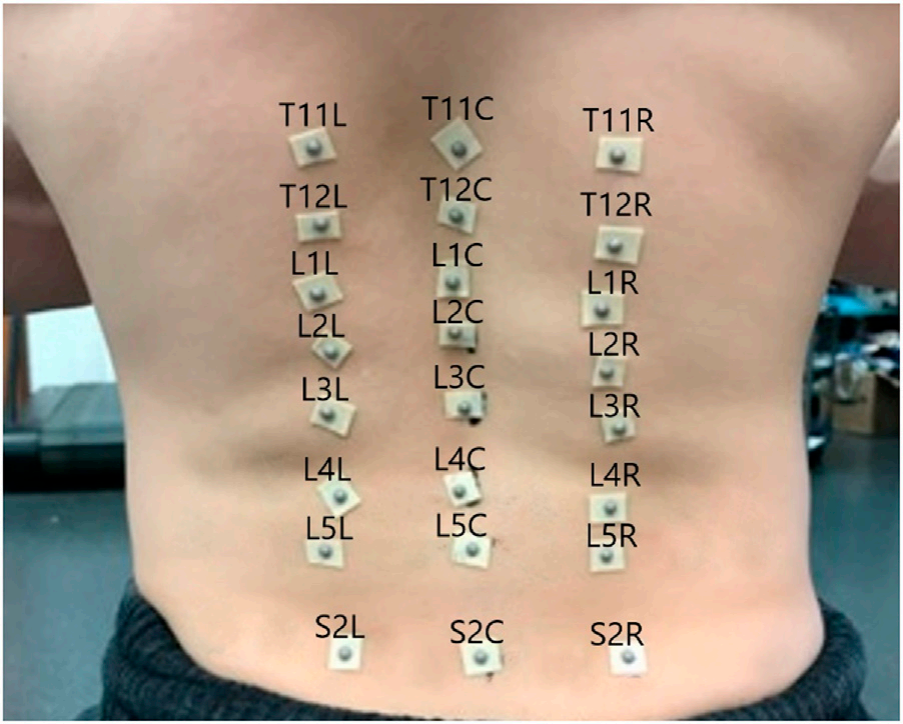
Motion capture marker placement, annotated with marker labels.

In the experiment subjects performed 3 repetitions of each of 17 diagnostic movements while being monitored by a 10-camera motion capture system (Qualisys Track Manager -Qualisys, Gothenburg). The motion capture tracked the positions of 24 hemispherical 4 mm retroreflective markers (B&L Engineering) on the subjects’ backs, placed in the locations shown in Figure 1, at 100 Hz. To locate the markers, a licensed physical therapist palpated the spinous processes of the T11-T12, L1-L5, and S2 vertebrae and marked them with ink. Markers were placed over the spinous processes as well as 1.75 inches to either side of each spinous process. Pilot testing indicated inadequate adhesion to the skin with the markers alone, so each marker was mounted on double sided toupee tape for extra adhesion.

Diagnostic movements were chosen based on previously published literature including uniplanar movements (Kondratek et al., 2007; Laird et al., 2014), combined movements (Edwards, 1979; Monie et al., 2015), multiplanar trunk kinematics during lifting tasks (Marras et al., 1993; Marras et al., 1995b; Knechtle et al., 2021), and dissociated lumbar/pelvic movements (Alrwaily et al., 2017), and “up and go” task (Pourahmadi et al., 2019). Movements were reviewed with each participant before data collection began. A researcher explained and demonstrated each diagnostic movement, and the participant practiced it at least once before data collection. Participants were instructed to move at a speed that was comfortable for them. Research suggests that when subjects move at a natural speed, more consistent motion characteristics are captured (McGregor and Hughes, 2000). General instructions were given that for each diagnostic movement, subjects were to move as far as was comfortably possible, but not to exert themselves. A trained physical therapist was on site to ensure adequate performance of each diagnostic movement prior to data collection. The diagnostic movements and their subject instructions are presented in Table 2.

**TABLE 2:** Instructions for each diagnostic movement, along with the numbers of subjects and trials included for analysis after removing movement samples with excessive marker tracking errors.

Diagnostic movement	Instructions	Subjects included in analysis	Trials included in analysis
Flexion	Bend as far forward as you can comfortably reach; slight flexion of the knees is allowed	25	74
Flexion-Left	Bend forward while rotating to the left	27	78
Flexion-Right	Bend forward while rotating to the right	27	79
Flexion-Box	Bend forward and pick up an empty box placed squarely in front of you; slight flexion of the knees was allowed	27	79
Flexion-Box-Left	Bend forward and pick up an empty box placed directly left of you; slight flexion of the knees was allowed	26	73
Flexion-Box-Right	Bend forward and pick up an empty box placed directly right of you; slight flexion of the knees was allowed	28	81
Extension	Bend as far backward as you can comfortably reach	25	68
Extension-Left	Bend backward while rotating to your left	25	68
Extension-Right	Bend backward while rotating to your right	25	73
Rotation-Left	Rotate your trunk toward your left side while maintaining pelvis position (facing anteriorly)	26	77
Rotation-Right	Rotate your trunk toward your right side while maintaining pelvis position (facing anteriorly)	26	65
Side Bending-Left	Bend laterally to your left while maintaining a forward-facing trunk orientation	26	78
Side Bending-Right	Bend laterally to your right while maintaining a forward-facing trunk orientation	26	75
Exercise Ball—Flexion	Sit on an exercise ball with an erect back posture, then bring your hips forward (tilting pelvis posteriorly), while maintaining the vertical orientation of your upper torso	28	82
Exercise Ball—Extension	Sit on an exercise ball with an erect back posture, then bring your hips backward (tilting pelvis anteriorly), while maintaining the vertical orientation of your upper torso	27	78
Exercise Ball—Flexion-Extension	Sit on an exercise ball with an erect back posture, then bring your hips first forward then backward while maintaining the position and vertical orientation of your upper torso	27	74
Up and Go	Sit on a short stool, then rise without the assistance of your hands and walk several steps forward	28	81

Each subject completed three repetitions of each of the 17 diagnostic movements with the whole cohort performing a total of 1,428 total motion samples. For cases in which the cameras lost track of markers gaps were filled via Qualisys Track Manager’s gap filling tools if the gap was less than 30 frames. In cases where excessive trajectory changes were detected, marker labels were examined and corrected if marker swapping had occurred. Motion samples which failed to capture all markers in the array, or which had excessive trajectory errors caused by large discontinuities (greater than 30 frames) or accelerations above Qualisys Track Manager’s default acceleration threshold of 150 m/s^2^ that were not caused by rectifiable marker misidentification, were omitted from the final analysis. The analysis included 1,283 motion samples with between 65–82 (avg 75) total trials for each diagnostic movement, with no movement having data from fewer than 25 subjects (Table 2).

### 2.3 Data processing

The positional data for each marker were processed using MATLAB R2020a. Data were aligned using the first significant peak in the primary plane of each diagnostic movement. MATLAB’s *findpeaks* function was used to find the indices of each peak in the trials. The largest peak in the primary plane of motion was considered as the central landmark in each motion, and all trials were aligned according to this. Extraneous data were cut off at the start and end of each trial by finding the first and last point in any planes during a trial that the velocity of one segment reached 5% of peak velocity. No data normalization outside of this alignment was performed.

**FIGURE 2:**
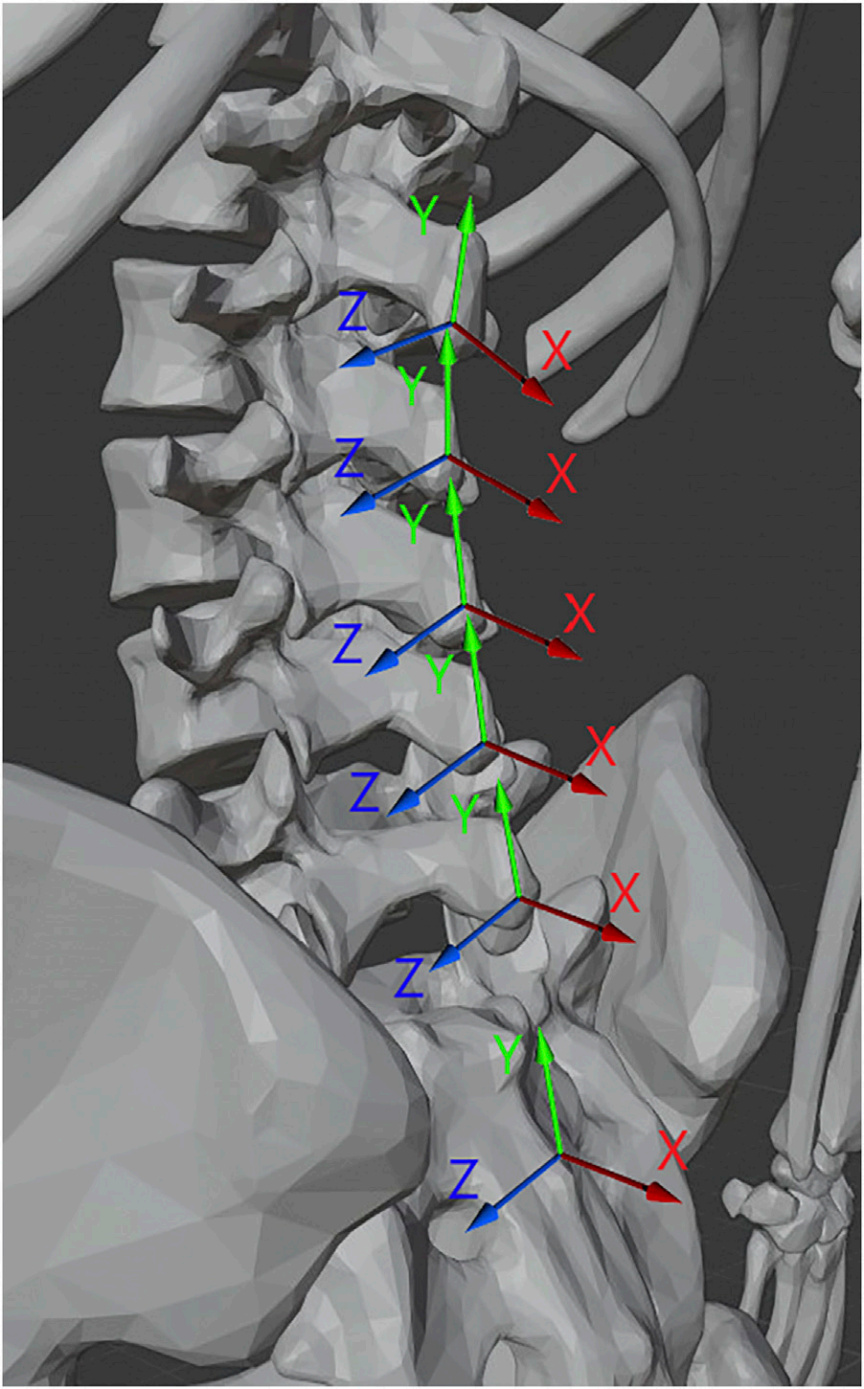
Illustration of the normal (X), coincident (Y), and binormal (Z) vectors for the functional segments of the spine. 3D Model credit Dario Baldi, Thingiverse.com, used with permission.

The positional information of the markers was first filtered using a third order Savitzky-Golay filter with a window size of 151 frames (corresponding to 1.5s of data based on the 100 Hz sampling rate), and then used to generate seven sets of local coordinate axes: one axes for each pair of markers indicating a functional segment between S2 and T11. These axes included an X vector normal to the back running anterior to posterior from each functional spinal unit (FSU), a Y vector running inferior to superior, and a binormal Z vector pointing medial to left lateral. These vectors were calculated for each frame of collected data in each trial as follows:

The coincident vector for each functional segment, shown as the green axis labeled Y in Figure 2, was defined as the vector from the marker over the inferior spinous process of the segment to the marker over the superior spinous process (see Eq. 1). All vectors for these axes were converted to unit vectors. For example, for the L4/L5 FSU the coincident vector is defined such that L4C is the position vector of the motion capture marker located on the spinous process of L4 (see Figure 1). All vectors were converted to unit vectors that correspond to the appropriate direction.

$$\vec{L_{5Y}}=\left(\vec{L_{4C}}-\vec{L_{5C}}\right)$$ (1)

The normal vector, shown as the red axis labeled X in Figure 2, was determined for each segment between T11 and S2 by taking the cross product of the vector from the marker to the left of the bottom-most spinous process of the segment to the marker to the right of the same and the coincident vector of the segment as shown in Equation 2.

$$\vec{L_{5X}}=\left(\left(\vec{L_{5R}}-\vec{L_{5L}}\right)\times \vec{L_{5Y}}\right)$$ (2)

The binormal vector of the segment, shown as the blue axis labeled Z Figure 2, was calculated as the cross product of the coincident and normal unit vectors as shown in Equation 3.

$$\vec{L_{5Z}}=\left(\vec{L_{5Y}}\times \vec{L_{5X}}\right)$$ (3)

From these axes, illustrated in Figure 2, three angles of rotational information relative to the bottom-most marker pair (over the sacrum) were calculated for each segment. Each angle was projected onto a standard radiographic plane (sagittal, coronal, axial) with the L5-S2 coordinate system acting as the reference global coordinate system. The *Flexion* angle (θ_F_) was calculated as the angle between the normal vector of the segment from the S2 skin marker to the L5 skin marker and the normal vector of the segment in question, using the binormal vector of the L5-S2 region as the axis.

$$\theta_{F}=\mathrm{asin}\left(\vec{L_{5Y}}∙\hat{i}-\vec{S_{2Y}}∙\hat{i}\right)$$ (4)

The *Lateral Bending* angle (θ_LB_) of each segment was calculated as the angle between the coincident vector of the L5-S2 region and the coincident vector of the segment in question, using the normal vector of the L5-S2 region as the axis.

$$\theta_{LB}=\mathrm{asin}\left(\vec{L_{5Z}}∙\hat{j}-\vec{S_{2Z}}∙\hat{j}\right)$$ (5)

The *Rotation* angle (θ_R_) was calculated as the angle between the binormal vector of the L5-S2 region segment and the binormal vector of the segment in question, using the coincident vector of the L5-S2 region segment as the axis.

$$\theta_{R}=\mathrm{asin}\left(\vec{L_{5X}}∙\hat{k}-\vec{S_{2X}}∙\hat{k}\right)$$ (6)

Each angle was offset so that the neutral standing position of the subject corresponded to a zero angle for each axis of the segments. From the zero angle, the maximum and minimum angles of rotation in each axis were found for each trial.

From the angular position data the time-series of the angular velocity of each segment was calculated using MATLAB’s *gradient* function and the sampling rate of the data (see Equations 7, 8). This was done using a central difference numerical differentiation as shown in Eq. 5 where P is a generic angular position vector over time, and F is the framerate (i.e., 1/F is the time increment between frames).

$$\nabla \vec{P}=\frac{\partial \vec{P}}{\partial x}i+\frac{\partial \vec{P}}{\partial y}j+\frac{\partial \vec{P}}{\partial z}k$$ (7)

$$\frac{\partial \vec{P}}{\partial x}≅\frac{\vec{P}\left(x+\frac{1}{F}\right)-\vec{P}\left(x-\frac{1}{F}\right)}{2\left(\frac{1}{F}\right)}$$ (8)

The angular acceleration was calculated similarly, using the *gradient* function on the angular velocity data.

### 2.4 Statistical analysis

Demographic data were collected for all participants, and one-way ANOVA tests were performed using MATLAB’s anova1 function to look for differences in segmental end RoM in each plane of motion for each FSU (i.e., 18 outcome variables) by gender, height, weight, and age. To check normality, Anderson-Darling tests were performed on end RoM data. Using a Bonferroni correction factor of 18 indicated that 61.8% of the 306 end RoM vectors were normal, which is reasonable based on sample size of the data (Blanca et al., 2017). End RoM data were calculated based on the point of maximum angular deflection for each individual segment. Average angular velocity data for ascending *versus* descending segmental motion were considered paired data and were analyzed using a multivariate approach via Hotelling’s T-squared tests. This testing methodology provided a more holistic view of the kinematics by considering all six FSU’s at the same time.

Bonferroni correction factors were used to account for the large number of comparisons. Each diagnostic movement was considered independent from one another, and a Bonferroni correction factor of 18 (*α* = .0028) was used during two-sample T-tests comparing each six FSU’s in each of the three planes of motion when comparing ascending *versus* descending motion or movements performed to the left *versus* the right.

### 2.5 Verification

Numerical simulations were undertaken to approximate the effects of marker misplacement and STAs. Marker misplacement was simulated by altering the starting position of a central marker in the *Y* and *Z*-axes in a Box_L movement. The Box_L movement was chosen because it is a triplanar motion that elicits kinematic activity in all three planes. Soft tissue artifacts were simulated by a ramped displacement from to the peak of a Flexion movement and back.

## 3 Results

### 3.1 End range of motion

#### 3.1.1 Symmetry

**FIGURE 3:**
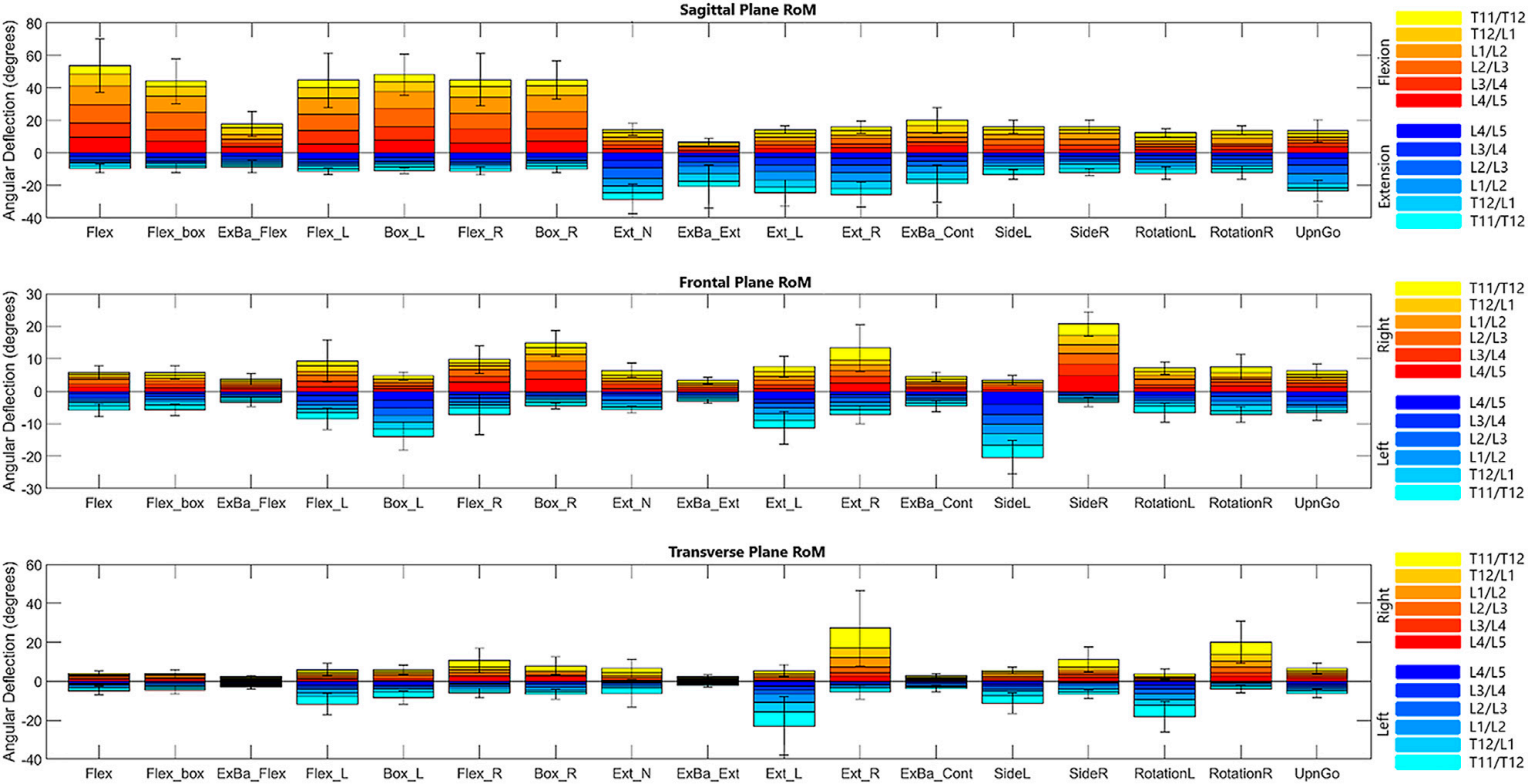
Bar graph of range of motion means with standard error bars for the composite lumbar region in each diagnostic movement. It is important to note that these values depict the maximum ROM for each segment throughout the entire movement.

End ROM for each segment are reported in Figure 3 for each diagnostic movement performed. The data indicate that there was a high degree of symmetry between left/right versions of each diagnostic movement (i.e., end ROM for movements performed to the left or the right were not significantly different in any plane when comparing using an adjusted alpha value of .05/18, or .0028). While none of the values reached statistical significance with this conservative alpha, the range for the 18 segmental level *p*-values calculated for each of the five left/right diagnostic movements ranged from .0048 to .94. Though not reaching statistical significance, four of the ten *p*-values that are below .05 are resultant from potential motion discrepancies the L4/L5 segment experienced in the frontal and transverse planes. Composite lumbar ROM was examined for differences as well, with the only meaningful difference in left/right diagnostic movements being in the sagittal plane between flexion left and right movements (*p* = .0048). All other diagnostic movements showed no statistical differences in composite lumbar range of motion (*p* = .073 to *p* = .916).

#### 3.1.2 Motion coupling

Due to geometrical properties of the vertebra and as expected, diagnostic movements performed in the transverse or coronal planes (i.e., rotation; lateral bending) also exhibit coupled motions in the coronal or transverse planes, respectively. For example, while performing rotation left and right, subjects exhibit an average of 1.3° of motion in each segment in the coronal plane. Similarly, lateral bending left and right exhibit an average of .6° of motion per segment in the transverse plane. Likewise, flexion exhibits an average of 2.4° of motion in the transverse plane and 2.5° of motion in the coronal plane, indicating coupled motion throughout the movement. A table included in the Supplementary Material includes data as percent coupled values similar to those in a systematic review on lumbar kinematics published by Widmer et al. (2019) for the three primary planes of motion in flexion/extension, lateral bending left and right, and rotation left and right.

### 3.2 Dynamic range of motion

Time dependent segmental motion data are reported as.

1. Position over time
2. Average angular velocity
3. Peak segmental angular velocity
4. Peak segmental angular acceleration

**FIGURE 4:**
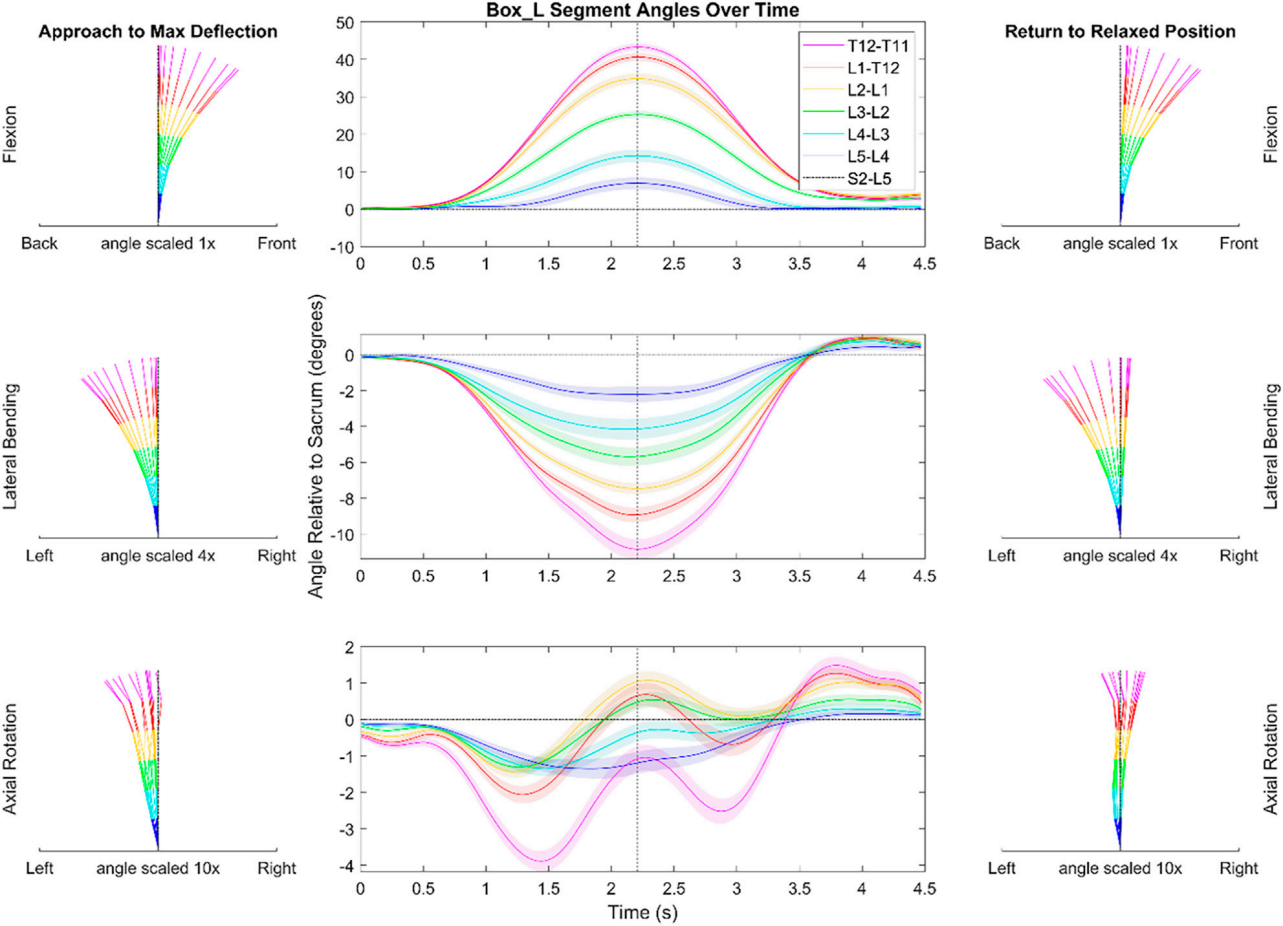
Dynamic range of motion data for the functional exercise of picking up a box to the left. The center graph shows the average motion of each segment across each trial and each subject. The shaded regions indicate the standard error associated with each segment. The dotted black vertical line indicates the peak that was used during data alignment. The kinematic visualizations on the left depict the subjects going towards end range of motion with even time spaces between each line. The kinematic visualizations on the right are similar to those of the left but depict the subjects’ movement back to a resting position. Note the scaling is different for motion in each plane. The shaded regions indicating variation for the center panels reflects the standard error of the mean.

**FIGURE 5:**
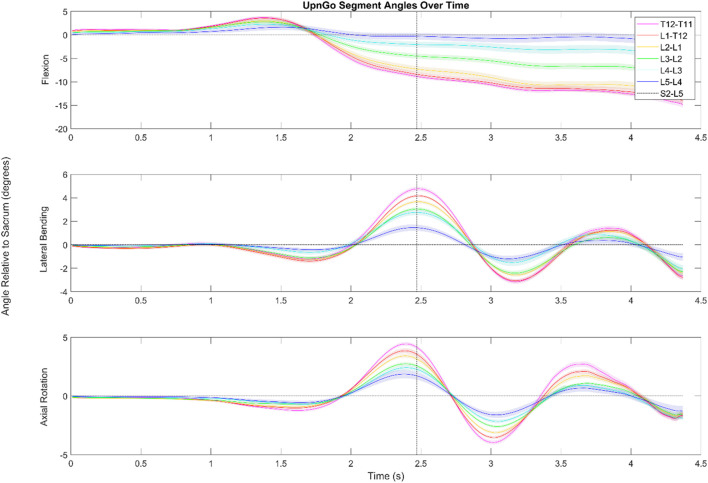
Dynamic range of motion data for the functional exercise standing up from a stool and walking. The shaded regions indicate the standard error associated with each segment. While the data look somewhat noisy for flexion/extension, this is due in large part to having aligned the data by the first foot step (as shown by the dotted black line) of the subject. Subjects were not instructed to lead with a particular foot, and in cases where subjects led with their left foot, the data was flipped to better visualize the aggregate data.

**FIGURE 6:**
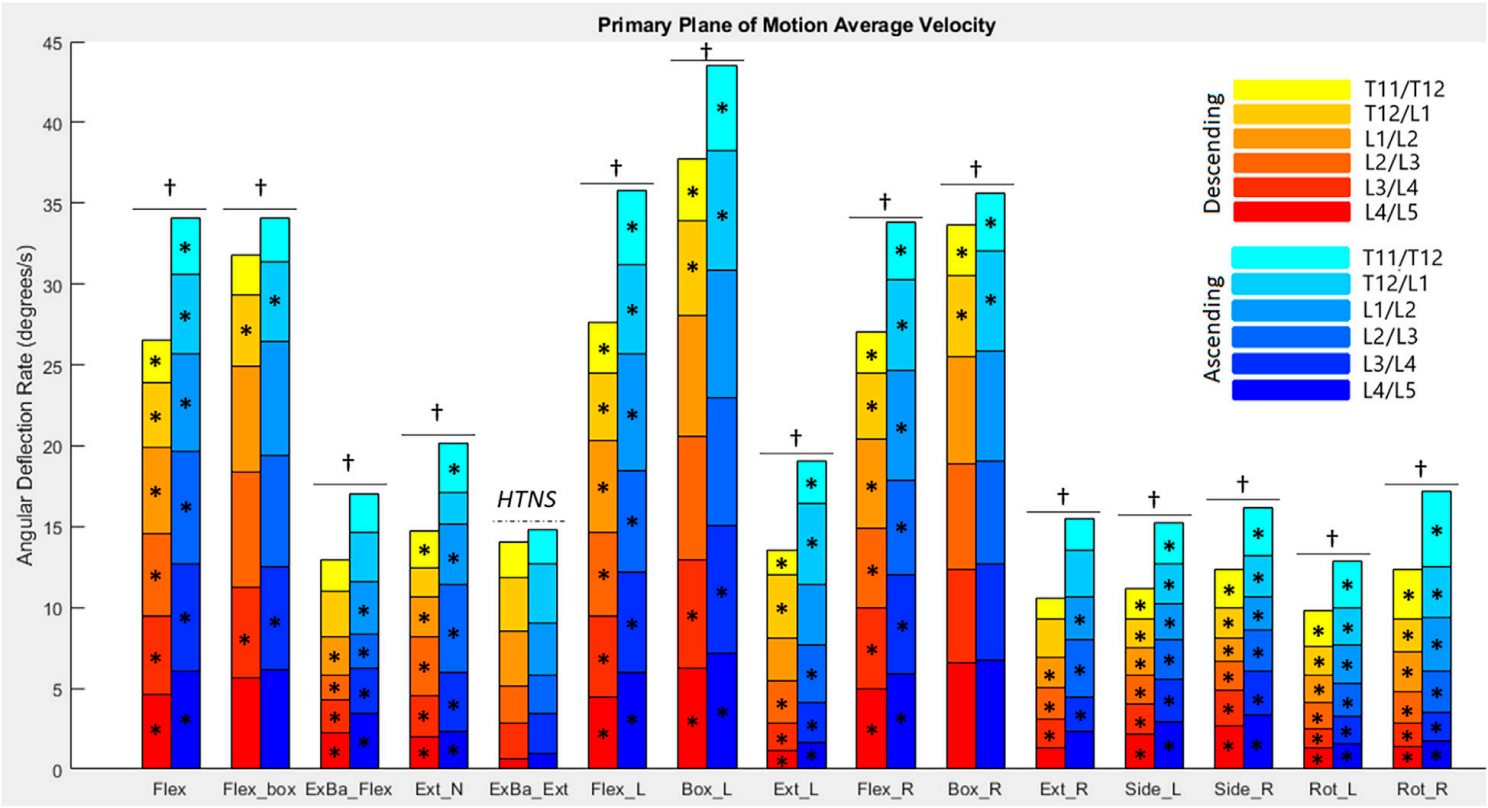
Average angular velocities for each diagnostic movement reported by segment. Hotelling’s T-squared testing showed all diagnostic movements except exercise ball extension were significantly different in their ascending vs. descending motions (α = .0038). The total angular velocity of the lower spine was significantly different (α = .05) when looking at descending vs. ascending motion for all diagnostic movements except exercise ball extension (as denoted by a dagger). Significance at the segmental level is denoted by asterisks (α = .0083).

**FIGURE 7:**
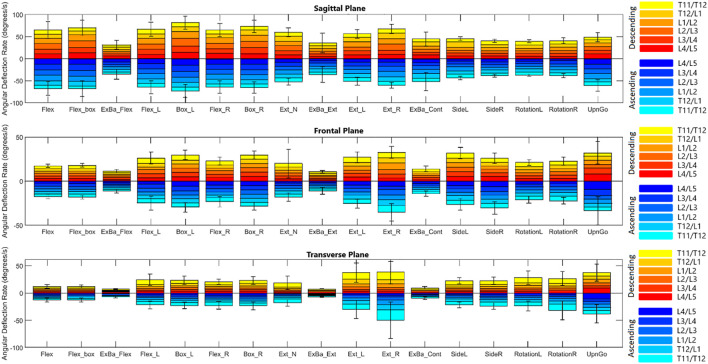
Peak angular velocities for each diagnostic movement reported by segment.

**FIGURE 8:**
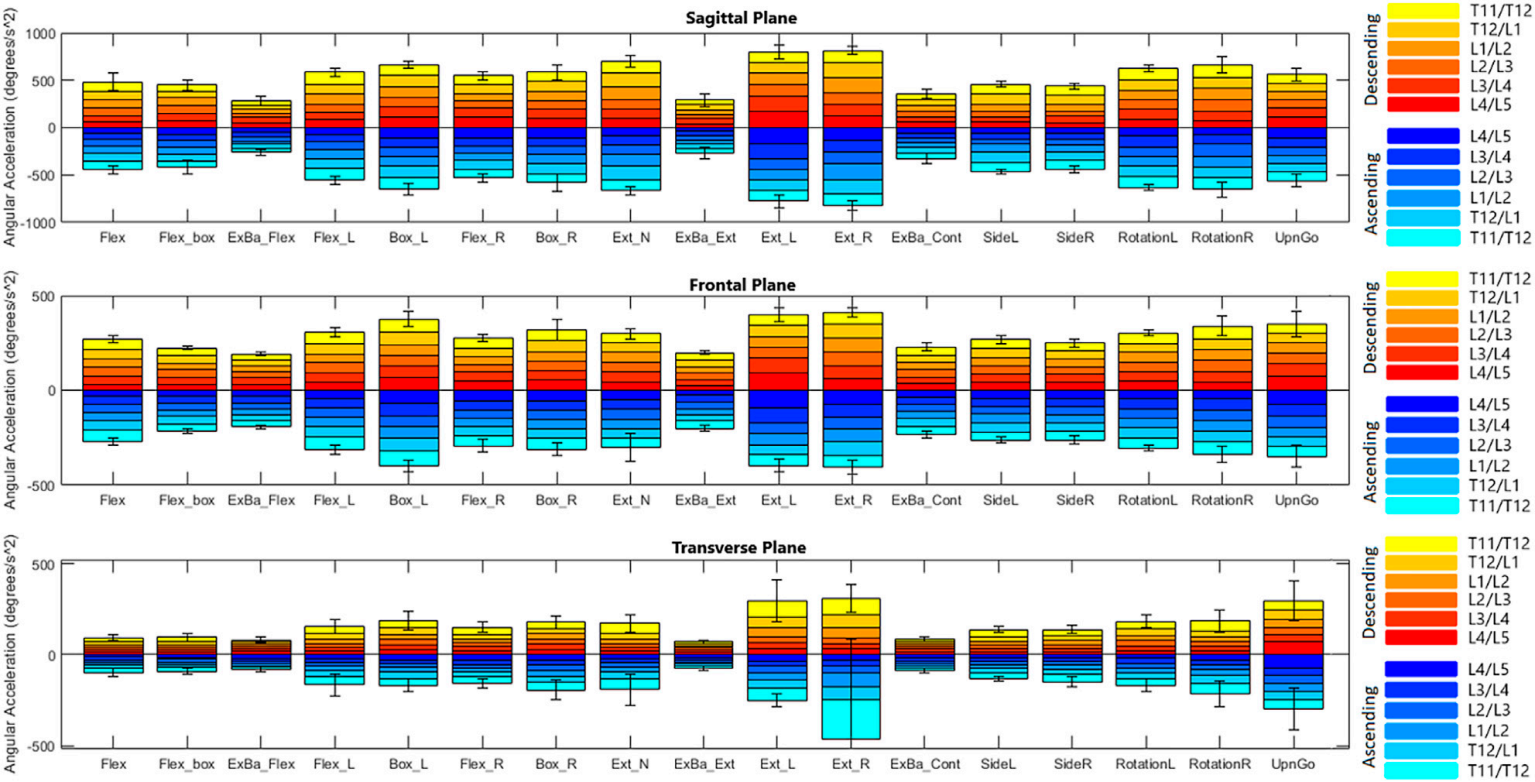
Peak angular accelerations for each diagnostic movement reported by segment.

Figures 4, 5 depict a dynamic graphical representation of the motion of each segment in each plane throughout two of the 17 diagnostic movements: *Box Left* and *Up and Go*. Similar figures for each of the other 15 diagnostic movements can be found in the online supplement to this article. Figure 6 reports the average angular velocity of 13 diagnostic movements for each segment in the primary plane of motion during travel to maximum deflection and travel back to starting position. Consistent with previous studies, we are using the terminology descending (outward) and ascending (return) motion (McGregor et al., 1995; Consmuller et al., 2012a). Figures 7, 8 depict peak angular velocities and accelerations for each segment in a bar graph for each diagnostic movement.

#### 3.2.1 Box left

In picking up a box positioned on the left as depicted in Figure 4, there was less flexion in the T11/T12 (3.2°) segment when compared to the other FSUs (5.3–10.8°). During ascending motion in this movement, subjects overshot their original starting position in the transverse and coronal planes by an average of 1.2 and .9°, respectively. Additionally, in the transverse plane, the data indicate a semi-periodic motion in the thoracolumbar joint (T11-L2). The peak and trough values for the T11/T12 segment are 3.9 and 1.3°, respectively. The equal time spaces between the (Figure 4A) kinematic visualizations depict the maximum accelerations at the start of the diagnostic movement and the maximum decelerations at the end of the diagnostic movement.

#### 3.2.2 Up and go

During an Up and Go test as seen in Figure 5, the subjects exhibited periodic motion in the lumbar spine as they walked forwards. The greatest ROM in this periodic motion in the transverse and coronal planes is exhibited in the L4/L5 segment and the smallest is exhibited in the L2/L3 segment.

During the Up and Go test, subjects start with slight flexion in the lower spine as they try to gain momentum to stand up. The lumbar spine then extends into a lordosis as they stand up straight. Since data are shown relative to the subjects’ starting position, the top graph depicts the lordosis of the lower spine once subjects are erect compared to when seated. The data indicate that in standing up, the L3/L4,L2/L3,L1/L2 segments go through a greater ROM in the sagittal plane than the L4/L5, T12/L1, and T11/T12 segments. The L3/L4 segment has an average maximum extension value of 4.8°, the L2/L3 segment has an average maximum extension value of 2.9°, and the L1/L2 segment has an average maximum extension value 1.8°.

Other points of interest in Figure 5 include the offset peaks of the ROM in the transverse and coronal planes. Range of motion in the transverse plane reached local minima and maxima .17 s before the peak in the coronal plane. Subjects also tended to exhibit small amounts (<2°) of lateral bending and axial rotation in the lower spine as they stood up.

#### 3.2.3 Average angular velocity

Figure 6 reports the average angular velocity of each segment in the primary plane of motion (sagittal for flexion, extension, and box diagnostic movements, frontal for lateral bending, and transverse for axial rotation) travelling towards maximum deflection and returning from maximum deflection. The continuous exercise ball diagnostic movement along with the Up and Go test were removed from this analysis, since they are non-cyclic motions. Hotelling’s T-squared testing was performed to compare the two directions of motion (ascending and descending) and found all diagnostic movements except for exercise ball extension had significant differences (*p* = 8.07e-14 to *p* = 0.00033) between the directions of movement for primary plane motion in each diagnostic movement. Segmental level t-tests were performed for all motions that had significant Hotelling’s T-squared test values. A Bonferroni correction factor of six was used to account for testing each segment (*α* = .0083). Sixty-three of seventy-eight measurements of the segments have significantly different speeds going to maximum deflection *versus* returning with *p*-values ranging from 2.33e–12 to .0073. In addition to segmental motion, composite lumbar spine speed was compared as well, with all 14 motions returning low *p*-values (5.63e–17 to .0176) and reaching significance at α = .05.

#### 3.2.4 Peak angular velocity and acceleration

Figures 7, 8 report peak angular velocity and acceleration. Peak angular velocities did not follow the same trend of being greater during ascending motion. No meaningful differences in peak descending and ascending velocities were found in any diagnostic movements. While not reaching statistical significance using a Bonferroni correction factor of 18 (*α* = .0028), there were 11 segments that had *p*-values below .05 in 8 different diagnostic movements (*p* = .01 to .048). Most of these segments are a single segment of a single plane in a single diagnostic movement and do not point to an area of further potential interest. However, extension right, box left, and box right all had motion of the L4/L5 FSU in the transverse plane that had *p*-values below .05 when comparing descending vs. ascending motion.

When comparing symmetrical diagnostic movements, the L4/L5 segment moves at a higher speed when the box movement is performed to the left rather than to the right (*p* = .0019). While this is the only segment at which the peak velocity difference between right and left diagnostic movements reaches significance at *α* = .0028, there are six other segments in different planes and diagnostic movements that had *p*-values below .05 (*p* = .007 to .042). When examining peak acceleration differences between symmetrical diagnostic movements, there were also 8 other segments in different planes and diagnostic movements that had *p*-values below .05 (*p* = .0048 to .049). Low *p*-values for these diagnostic movements were scattered with the exception of the box left *versus* box right movements, particularly in the frontal plane. The T11/T12, T12/L1, L1/L2 segments trended to moving faster in the frontal plane during box left movements than during box right (*p* = .010 to .033). The T11/T12, L1/L2, L2/L3, and L3/L4 segments trended towards accelerating faster in the frontal plane during box left movements than during box right (*p* = .0048 to .042).

## 4 Discussion

### 4.1 Demographics

In performing ANOVA analyses to examine the influence of height, weight, gender, and age on the results, it was found that none of these factors caused significant differences in the primary plane of a motion except for during the Up and Go test, where the L4/L5 segment moved differently in females *versus* males (*p* = .042). While a larger cohort may produce more definite results, this difference supports Pourahmadi et al. (2020), who found that males and females exhibited differences in ROM at a segmental level, but not at the whole lumbar level, during a sit to stand clinical exercise (Pourahmadi et al., 2020). Additionally, age has been shown to affect end ROM in previous work (Consmuller et al., 2012a), and BMI has been shown to be correlated to soft tissue thickness, which likely also influences spinal kinematics (Sistrom et al., 1993; Kawchuk et al., 2011). Specifically, variation in body fat or muscle volume may affect these spinal kinematic measurements, whether from a restriction in subject mobility, or whether from greater soft tissue thickness under the markers, particularly the left and right marker columns. The inability to see effects from these in our data is likely a result of the small population made of primarily college-aged participants.

### 4.2 Segmental end range of motion

In comparing segmental end ROM in symmetric diagnostic movements (such as box left vs. box right), the end ROM for all diagnostic movements performed to the right *versus* the left showed no statistical differences in the primary planes of motion (*p* = .0048 to .94. This is unsurprising due to the inclusion criteria for subjects with no history of back pain and is consistent with previous findings (Gombatto et al., 2008). However, it may be of interest to further examine the motion of the L4/L5 segment in the frontal and transverse planes. During flexion left *versus* flexion right, the L4/L5 segment was most different of all segments in end ROM in the frontal plane (*p*-value = .0048). In three of the other four symmetrical diagnostic movements, this segment also had low *p*-values when comparing transverse plane end ROM (*p* = .021 to .035). While these values do not constitute statistical significance with the correction factor used, it still presents the question, is there is a meaningful difference in how this segment moves in these planes? Given that the symmetric box diagnostic movements reported a *p*-value of .076 for differences in end ROM in the frontal plane in the L4/L5 segment, it may be of worth to research these potential asymmetries further. It is notable that there were not any diagnostic movement pairs that had low *p*-values in more than one plane for the L4/L5 segment. This indicates a lower likelihood of compensatory motion in one of the other planes. Larger sample sizes or including data on handedness may be able to draw out further details that may have clinical significance. These motion differences could be of especial import as the L4-S1 region is more vulnerable to disc herniations, spondylolisthesis, and annulus-driven degeneration (Adams et al., 2015). It also has a tendency to show larger and a greater quantity of vertebral endplate signal changes (Jensen et al., 2009).

### 4.3 Motion coupling

The coupling of lateral bending with axial rotation in the lumbar spine has been well documented (Gercek et al., 2008; Kozanek et al., 2009). While some studies report coupling in flexion and lateral bending motions, the most commonly examined motion is axial rotation, in which the literature reports ranges from approximately 0.7–2.1 degrees of lateral bending in each segment when performing axial rotation to maximum end ROM (Shin et al., 2013). Our data are consistent with the overall values shown in previous studies investigating coupling motions in the spine in all planes [See Supplementary Material for percent coupling values to compare to Widmer et al. (2019)] When looking at coupling motion in the coronal plane while performing axial rotation, we found that the coupling in the L3/L4 and L4/L5 segments was in the same direction as the movement was performed. Higher segments tended to exhibit coupled motion in the opposite direction from these two lower segments. Most studies agree that the L3/L and L4/L5 segments couple in the same direction as the rotation occurs (Ochia et al., 2006; Fujii et al., 2007; Barnes et al., 2009; Kozanek et al., 2009), however some studies like Shin et al., suggest otherwise (Shin et al., 2013). Differences such as sample size, loading conditions, and instructional differences, such as arm position or seated *versus* standing rotation, likely account for the differences. In all, further research into coupling is needed to accurately phenotype healthy patterns of movement.

### 4.4 Dynamic segmental range of motion

#### 4.4.1 Box left

In picking up the box positioned on their left (see Figure 4), the subjects must rotate almost 90° to the reach the box and pick it up. However, the data show that this rotation does largely not occur in the lower spine. The largest angular deflection in a measured FSU is about 2.8° while most of the segments exhibit less than 1.5° of axial rotation. Therefore, the rotation necessary to perform this movement must come from other joints such as the subtalar joint, the hip joint, and/or the upper thoracic spine. Subjects with cLBP may accentuate this or similar trends to minimize motion in the lower back.

When comparing this box movement to flexion (see Supplementary Figure S1), the box movement takes approximately 1 s less to complete and does not exhibit the same plateauing shape in the sagittal plane as uniplanar flexion does. This is likely due to the influence of pelvic motion, commonly described as lumbo-pelvic rhythm during the motion of flexion. The lumbar vertebrae begin to move caudally to rostrally, and as they start to near end ROM, the pelvis becomes the primary driver of motion. Pelvic motion comprises the majority of the motion nearing full trunk flexion, but the spine also has larger end ROM enabled by this lumbo-pelvic rhythm. The extra sagittal end ROM in flexion compared to picking up a box is indicative of the lumbo-pelvic rhythm. Future work could provide further details on kinematics such as further understanding of in-phase *versus* out-of-phase motion for multiplanar movements or a more detailed look at the L/P ratio broken down by segment (Esola et al., 1996; Kim et al., 2013; Tafazzol et al., 2014; Laird et al., 2018; Ghasemi and Arjmand, 2021). Although it is not the focus of the present work, a closer investigation of additional time-sequenced coupled movement patterns (i.e., spinal rhythms) may provide mechanistic insights into the observed spinal motions.

During the diagnostic movement of picking up a box positioned on the left side, subjects tended to overshoot and laterally bend to the right upon returning to the starting position. This trend was common in many of the movements measured and has been shown in simple flexion tests as well (Sui et al., 2011; Consmuller et al., 2012a). It is possible that the subjects’ trunk right rotators, i.e., left external abdominal obliques, left lumbar multifidi, and right internal abdominal obliques, were activated too much or that their counterparts were not able to properly decelerate the movement, which was amplified by the weight of the box. In the similar diagnostic movement of performing flexion with axial rotation to the left, subjects had similar overshoot in the coronal plane (see Supplementary Material). However, the subjects took an additional .4 s to return to their neutral position after overshooting with the box.

#### 4.4.2 Up and go

During the Up and Go tests (see Figure 6), the maximum ROM of the periodic movement occurs in the lowest part of the spine. The movement of the pelvis in the coronal plane during the initial steps lead to a large deflection at the bottom of the spine that slowly is compensated as the spine makes up for the movement.

At the 2.5 s mark when the subjects start to walk, it is visible that the largest ROM in the frontal plane occurs at the L4/L5. The L3/L4 segment follows closely. This lateral bending to the right is necessitated by the left hip rising and the lumbar spine counter-side bending. The difference in peak timing during walking suggests that the spine does not rotate in the transverse and coronal planes at the same time, but rather individual muscles such as the rotatores and the erector spinae pull at different times throughout the gait cycle and cause the offset peaks.

We were unable to locate studies that have reported the kinematics of the timed Up and Go test, though a few studies have looked at the kinematics of a sit to stand test (Parkinson et al., 2013; Alqhtani et al., 2015; Christe et al., 2016; Hemming et al., 2018; Pourahmadi et al., 2020). Most of these studies look at total lumbar ROM, and our data fall within the previously reported values.

#### 4.4.3 Average angular velocity

In contrast to the majority of the diagnostic movements, we observed that several of the box lift and extension diagnostic movements did not exhibit any meaningful difference in descending and ascending average velocities in multiple spinal segments. We hypothesize that the extra weight of the box in the ascending motion slowed subjects down. Trends for flexion and extension disagree with the findings of Consmuller et al. (2012a) in which the average ascending velocity for flexion was greater than the descending velocity, while extension was significantly faster during the descending portion of the movement. It is likely that subject instructions played a role in this. In general subjects were instructed to move at a comfortable speed, however, some subjects in the present work were instructed to move slower during extension movements due to excessive marker dropout during their practice runs.

There is some disagreement in previously published work, with some authors reporting faster average descending velocities [e.g. (McGregor et al., 1995)], and others suggest that is not always true [e.g. (Marras and Wongsam, 1986)]. In the present study, it appears that certain subjects favor moving slower on the ascending motion than the descending motion. Discrepancies in past work may be due to biased samples or small sample sizes, though both the Consmuller et al. (2012b) and the McGregor et al. (1995) studies had over 200 participants.

Average velocities of the entire lower spine in flexion and extension had smaller values as compared with those reported in previous work (Marras and Wongsam, 1986; McGregor et al., 1995; Consmuller et al., 2012a). Consmuller et al. (2012b), Marras and Wongsam (1986), and McGregor et al. (1995) all report larger descending flexion velocities (with standard deviations well within the present work’s data) than the present work reports. Respectively, they report average descending angular velocities of 55, 48, and 30.3 deg/s compared to the present work’s 28.3 deg/s. Average ascending angular velocities also differed with reported respective values of 49, 36, and 51.3 deg/s compared to the present work’s 34 deg/s. Discrepancies in methodology should be also noted, such as the measurement system for the Consmuller study ran further up the thoracic spine, and the measurement systems used in the Marras and McGregor studies included data on the L5/S1 joint. Our measurements for axial rotation and lateral bending are also smaller than the data reported in the McGregor study.

In addition to differences in the spinal region measured, the protocol for calculating average velocity is different in the present work. Measurements in the Consmuller and McGregor studies are restricted to strictly the descending and ascending phases of the motion and ignore the peaks of the motion due to inconsistencies in how long subjects stayed at their maximal ROM. Since the present work also reports maximum angular velocity for each motion, it was decided to include the maximal ROM phase in the analysis to provide data on how these peaks affect average velocities. This inclusion of the maximal ROM phase where subjects slow down and change direction also greatly explains why the present work reports smaller average velocities than previous papers. It also denotes that there are grounds for a more direct comparison between our peak angular velocity data and the average velocity data in these previously published works.

#### 4.4.4 Peak angular velocity and acceleration

Peak angular velocities recorded appear to be higher than values reported in the Consmuller et al. (2012a) and McGregor et al. (1995) studies mentioned above when accounting for the number of spinal segments measured. In the Marras and Wongsam (1986) study above, subjects were asked to perform prescribed movements at a normal rate as well as at an accelerated rate. Recorded peak velocities of 63 deg/s for descending flexion and 65 deg/s for ascending flexion were well below the accelerated rate (108 deg/s descending, 76 deg/s ascending), but were still higher than the normal rate. While these values are similar, the same methodology discrepancies factor into data differences.

The significantly different descending and ascending average velocities compared to the descending and ascending peak velocities that showed no difference indicate that on average people either tend to take longer to start a diagnostic movement than to finish it or take longer slowing down at maximal deflection than speeding up at maximal deflection. In the latter case, the concentric contraction of the large back and abdominal muscles may be more explosive than the eccentric contraction of the muscles during these trunk movements. Another school of thought is that subjects are more cautious when descending to a new position, and less hesitant when returning to a recently held position. A further psychological factor could be due to watching demonstrations of the research assistants who may have unknown biases towards certain movement patterns.

This was a healthy cohort and symmetrical motion discrepancies were not expected. When looking at the results of the peak angular velocities and accelerations (see Figures 7, 8), it is of no surprise that there are generally no differences in the peak velocities and accelerations when performing a diagnostic movement to the right *versus* the left for a healthy group of people. However, it is interesting to note the asymmetries in the box movements when comparing descending and ascending velocities. One hypothesis is that the body’s dominant side has stronger muscles and the contractions of the external obliques of the dominant side provide the bulk of the power required to perform a rotation movement. In the current study, handedness was not recorded, however it could be of interest in future work. It is hypothesized that the extra weight of the box makes this a more pronounced difference and is therefore visible compared to the bodyweight only movements.

In comparing our measured values to those found in the literature, velocity and acceleration data are not widely reported at the segmental level in the lumbar spine. When comparing total angular velocities and accelerations however, these data fall within the range of previously reported total lumbar values (Consmuller et al., 2012a; Williams et al., 2014; Vaisy et al., 2015).

### 4.5 Measurement accuracy

#### 4.5.1 Comparative values

One purpose of this study was to assess the feasibility of measuring dynamic segmental kinematics of the lumbar spine during diagnostic movements using a traditional motion capture system. The present work indicates that quantitative estimates of kinematic measurements for each segment can be obtained in all three planes of motion. Accuracy of the measurements was addressed through comparisons with published data gathered through well-established methods of ROM measurement such as the use of bone pins or DFV for segmental motion, and inclinometers for end ROM.

Many systems have been used to measure active end ROM of the spine, but few have reported segmental motion or dynamic kinematics. Cobian et al. (2013) measured lumbar ROM and rates of excursion in subjects performing tasks of daily living using a motion capture system. They showed total lumbar excursion rates for daily movements to generally be well below a healthy person’s maximal excursion rates when the subjects performed simple flexion, lateral bending, and axial rotation movements (Cobian et al., 2013). Cook et al. (2015) measured cadaveric lumbar FSU ROM in an *ex vivo* environment using a motion capture system. They reported detailed segmental ROM for the lumbar region when testing flexion/extension, lateral bending, and axial rotation and compare their results with previous *ex vivo* and *in vivo* findings (Cook et al., 2015). However, their findings, along with other cadaveric studies (Cholewicki et al., 1996; Zirbel et al., 2013; Stolworthy et al., 2014), reported no time dependence in the motions, and do not capture the same details that measurements on living subjects would. Rozumalski et al. (2008) measured segmental ROM data during gait as well as flexion/extension, lateral bending, and axial rotation on a cohort of live subjects using bone pins and a motion capture system (Rozumalski et al., 2008). Our data compare favorably to the gold standard of bone pin measurements as performed by Rozumalski et al. Consmuller et al. (2011) and Suter et al. (2019) measured lordosis angles in the spine using a strain gauge system. Consmuller et al. measured segmental end ROM, and Suter et al. took continuous data on the average lordosis of the lumbar spine with both a motion capture system and the strain gauge system, but these measurements only examined sagittal plane motion (Consmuller et al., 2012b; Suter et al., 2020).

Our end ROM flexion and extension values gave similar results (5.1% larger than flexion and extension values that were measured using bone pins, and segmental end ROM measurements ranged from 2.4% to 24.1% different) (Rozumalski et al., 2008). Flexion and extension values from our trials also came within 10% of previous studies that used inclinometers or motion capture to measure end ROM in the lower spine (Cobian et al., 2013; Kolber et al., 2013). Values for lateral bending and axial rotation were less consistent with previous data, with total end ROM values ranging from being 40.0%–66.9% smaller.

Segmental end ROM ranged from 24.1% greater to 101.1% smaller than previously reported values (Rozumalski et al., 2008). Potential confounding factors besides skin artifacts include differences in instructions given to the subjects performing the diagnostic movements and reference points for the measurements. With the focus on functional movements, our subjects were to move as far as was comfortably possible, but not to exert themselves to go as far as possible. In addition, for movements such as axial rotation, subjects were instructed to keep their hips facing anteriorly which reduces FSU end ROM (Moreside et al., 2013). The studies with the compared ranges do not always give details on the instruction subjects received for these movements. Some of the reported differences may also be related to distinct subject demographics. For example, a study performed by Rozumalski et al. studied 10 healthy subjects but does not include data on the age of the subjects. It is possible that they had a different age demographic than the average age of 25 in this study.

#### 4.5.2 Marker placement effects

All skin-mounted marker measurements are susceptible to influence from the locational placement of the markers. Many studies have quantified this effect in various parts of the body (Della Croce et al., 2005; Gedet et al., 2007; McFadden et al., 2020; Severijns et al., 2021). Severijns et al. (2021) focused on the effect of marker placement in measuring spinal kinematics in subjects with adult spinal deformities. In their control population, they found that mediolateral palpation errors had significant correlations to BMI and soft tissue thickness. Inferosuperior palpation errors did not show the same correlations.

Numerical simulation was undertaken to examine the effects of marker misplacement on segmental kinematics. The L4C marker was shifted 5 mm in the -Z direction, 5 mm in the +*Y* direction, followed by a combination of 5 mm in both the Y and -Z directions.

**FIGURE 9:**
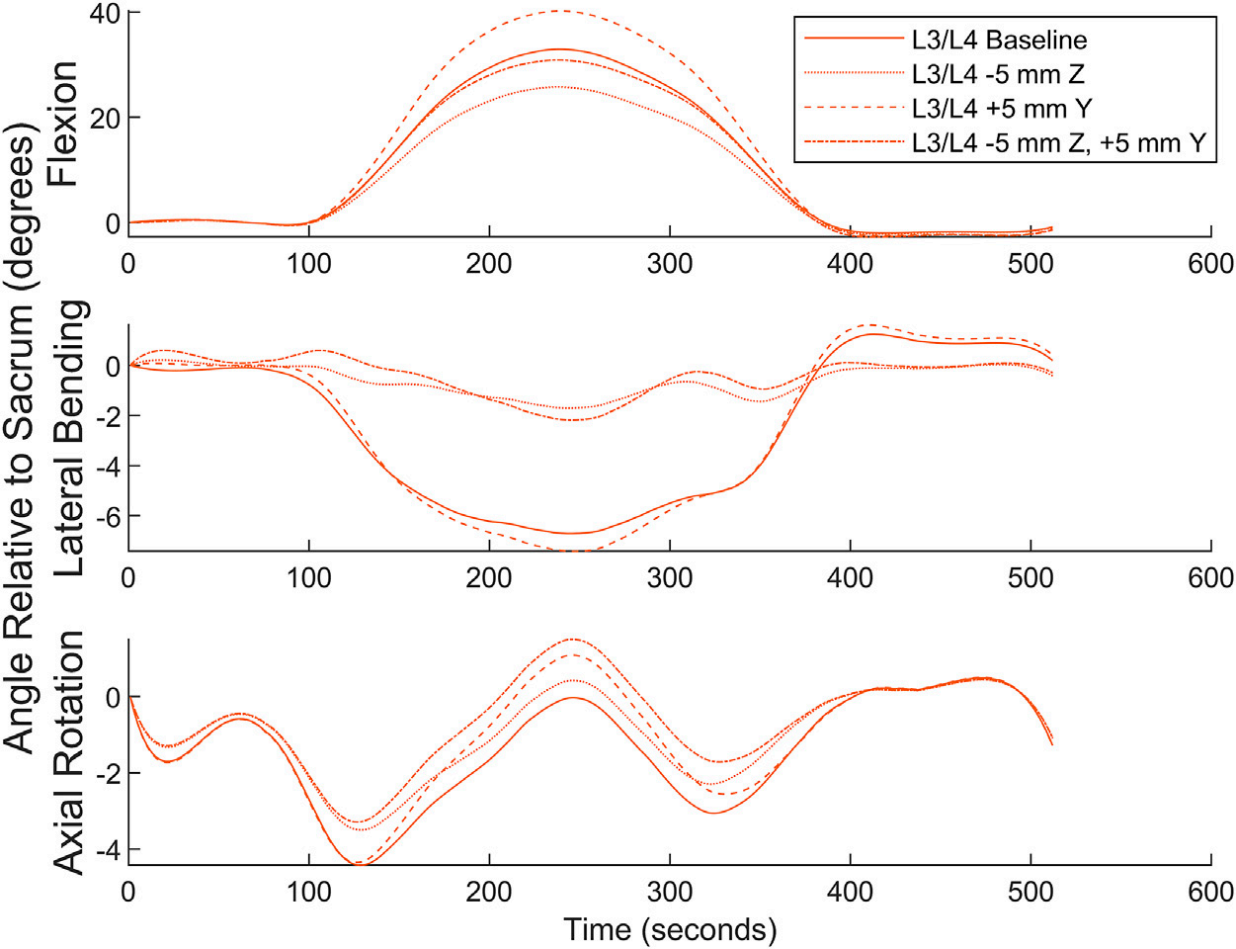
Numerical simulation of the effects of L4C marker misplacement in the Y and -Z directions.

Simulation for the superior segment affected by a central marker (see Figure 9) shows that the method is relatively insensitive to marker misplacement during Flexion and Axial Rotation but exhibits a higher sensitivity to marker misplacement (particularly in the *Z*-axis) during Lateral Bending. Central marker sensitivity was examined since the lateral markers were measured off the palpated central markers, and it is expected the greatest source of variation in both intra- and inter-operator reliability would originate from variations due to skill in palpating the spinous processes. A similar figure for the effects on the inferior segment can be found in the Supplementary Material.

The high-density marker set used in the present work provides an advantage in that calculation of segmental lumbar angles is possible (as opposed to a gross overall measure of total lumbar angle). However, the high-density marker set may magnify the local systematic error measures of those calculations in comparison to methods that examine total lumbar kinematics.

#### 4.5.3 Soft tissue artifacts

Soft tissue artifacts are an additional source of error. These artifacts include both motion artifacts caused by inertial forces as well as artifacts caused by the skin and underlying bones moving at different rates. The soft connective tissues such as the supraspinal ligament, subcutaneous layer (including adipose tissue), and cutaneous membrane that compose the low-stiffness connection between the spinous processes and the skin surface where the markers are located introduces a region for greater error that grows as the overall thickness of the soft tissue layers grow (Sistrom et al., 1993; Kawchuk et al., 2011). Soft tissue artifacts are both task dependent and subject specific (Leardini et al., 2005; Zemp et al., 2014). Zemp et al., and Morl et al., both measured marker shift on skin in relation to the spinous processes of lumbar vertebrae using vertical MRI machines (Morl and Blickhan, 2006; Zemp et al., 2014). Zemp et al. found an average skin shift of 7.1 mm for L1 through L5 in the inferior-superior direction in flexion with a maximum shift of 27.4 mm while Morl et al. found a median skin shift over the L3 and L4 spinous processes of .86 mm with a maximum shift of −9.86 mm in three different positions.

Zemp et al. also reports lumbar curvature as measured by markers and by MRI for L1-L5 and show average discrepancies of 17.1–21.8° depending on position (Zemp et al., 2014). Similarly reported discrepancies in the thoracic kyphosis (T3-T11) ranged from −8 to 4.7°. Their work states that foam tubes were attached to subjects’ backs to protect the markers while sitting against a backrest. However, the attachment method is not reported and may have affected the skin stretch in different postures (Cimino et al., 2018). In all, these works indicate that motion trends may be captured by optical motion markers, but they call for caution and awareness of the shortcomings in interpreting the results.

**FIGURE 10:**
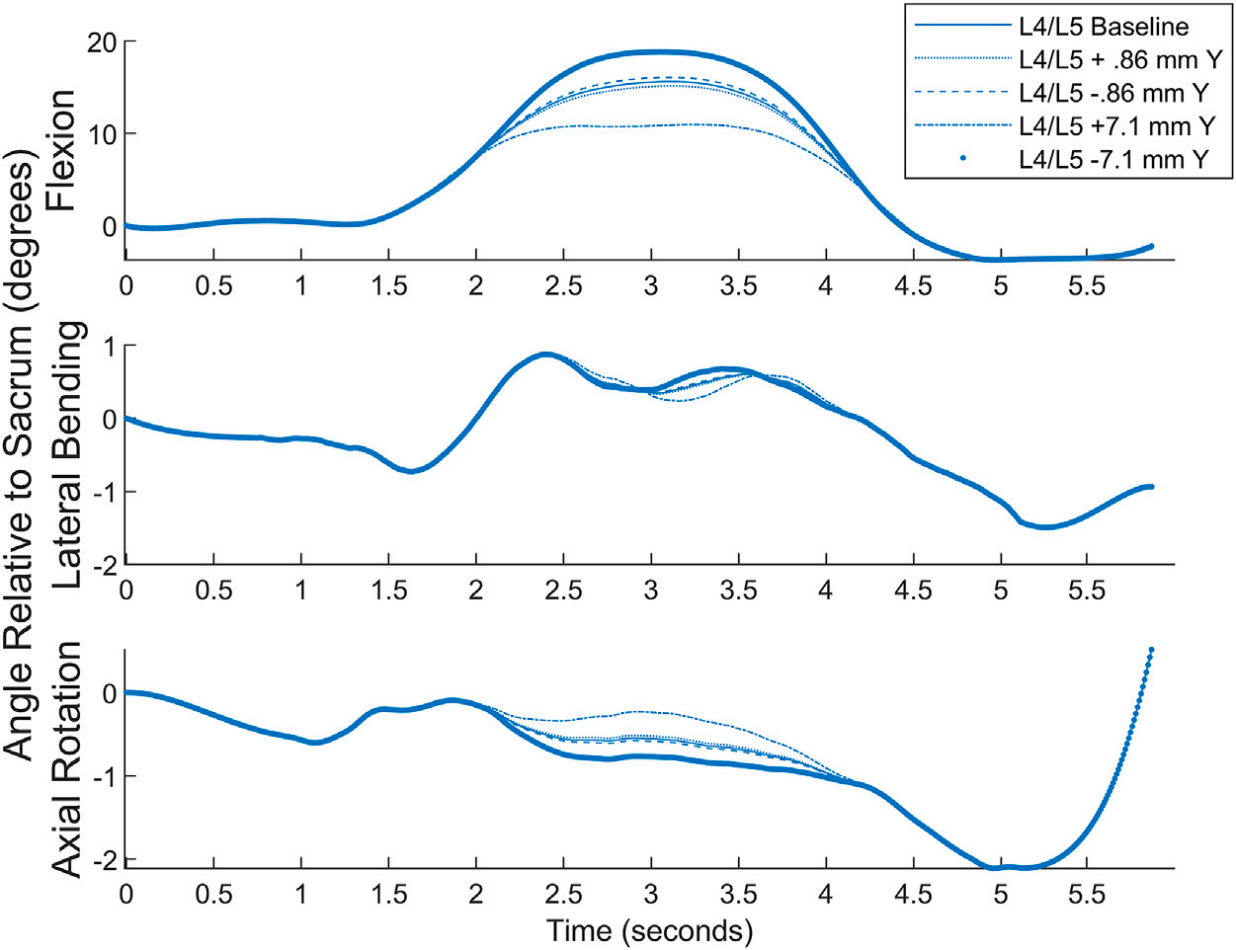
Simulated STA comparison showing approximate differences from measured values to different scenarios.

To estimate effects of STA’s on the present data, numeric estimations were made ranging from the 0.86 mm median to the 7.1 mm average inferior-superior STA’s cited above for flexion. Ramped deflections in both the positive and negative Y were applied to the L4C marker starting 1 s before and ending 1 s after the peak of the motion as found by MATLAB’s *findpeaks* function. Figure 10 depicts the results of this simulation. While this simulation does not account for any errors between the markers and the vertebrae in the initial orientation, it quantifies potential differences in true vertebral body motion compared to motion as measured by the markers. It is anticipated that similar changes would happen in other planes in other motions, but few spinal STA quantification data are available outside of flexion-extension.

#### 4.5.4 Effects of normalization

An important aspect of interpreting these data, especially for periodic motions, is understanding the normalization of the data. Picking the landmark about which to normalize the data can impact the results. Average range of motion at the normalization points are higher, and other areas tend to have lower values for the segmental range of motion. This is due to greater variations in subjects’ data the further away from the point of normalization. It is particularly notable in the periodic exercises Up and Go, and Exercise Ball Continuous. All other diagnostic movements were non-periodic and show less variation based on normalization point with uniplanar motions showing the least variation, and multiplanar motions showing slightly more variation based on the chosen normalization point.

#### 4.5.5 Effects of filtering

**FIGURE 11:**
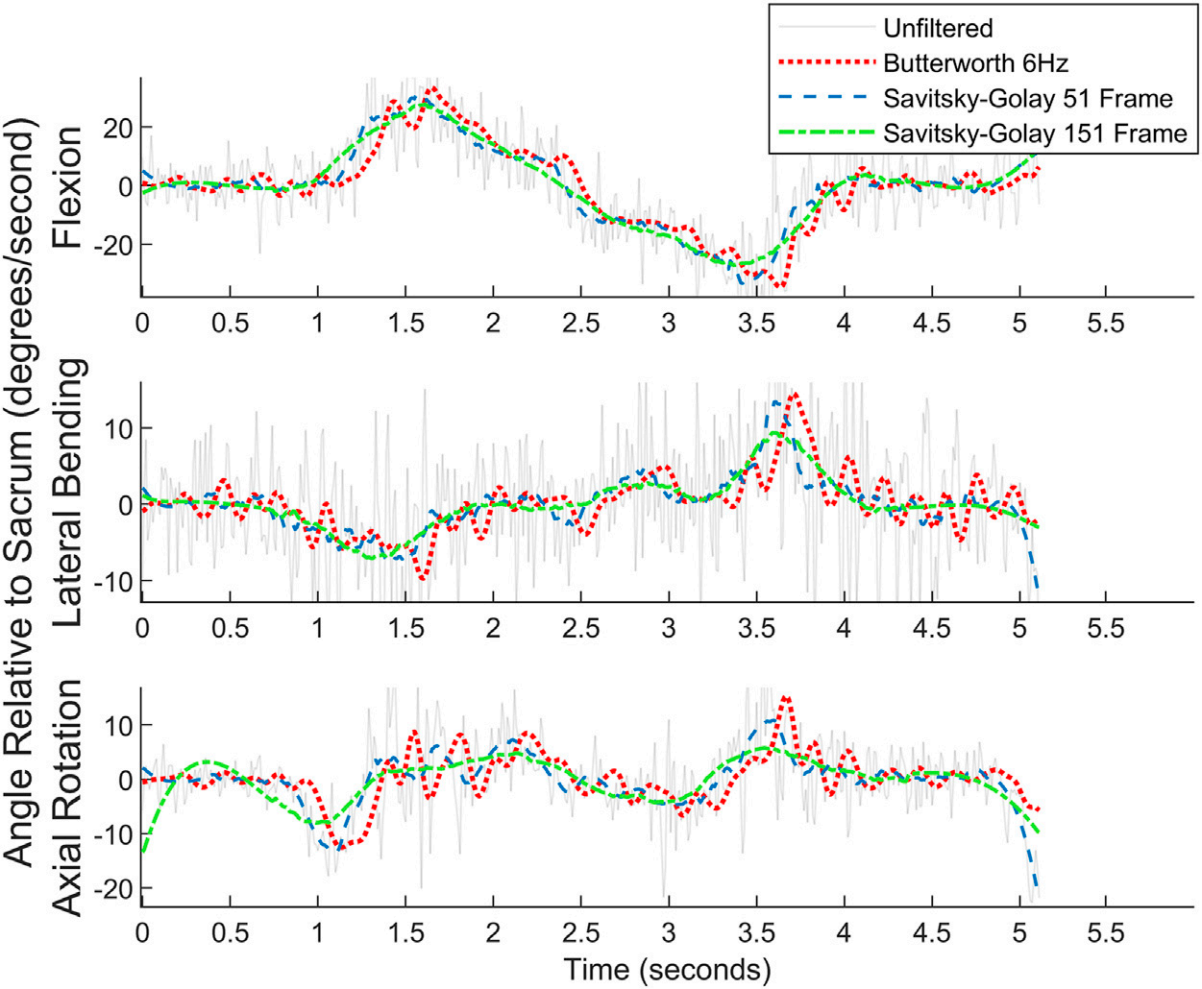
Angular velocity data for picking up a box on the left for one subject. Raw data are overlaid with the filtered data.

**FIGURE 12:**
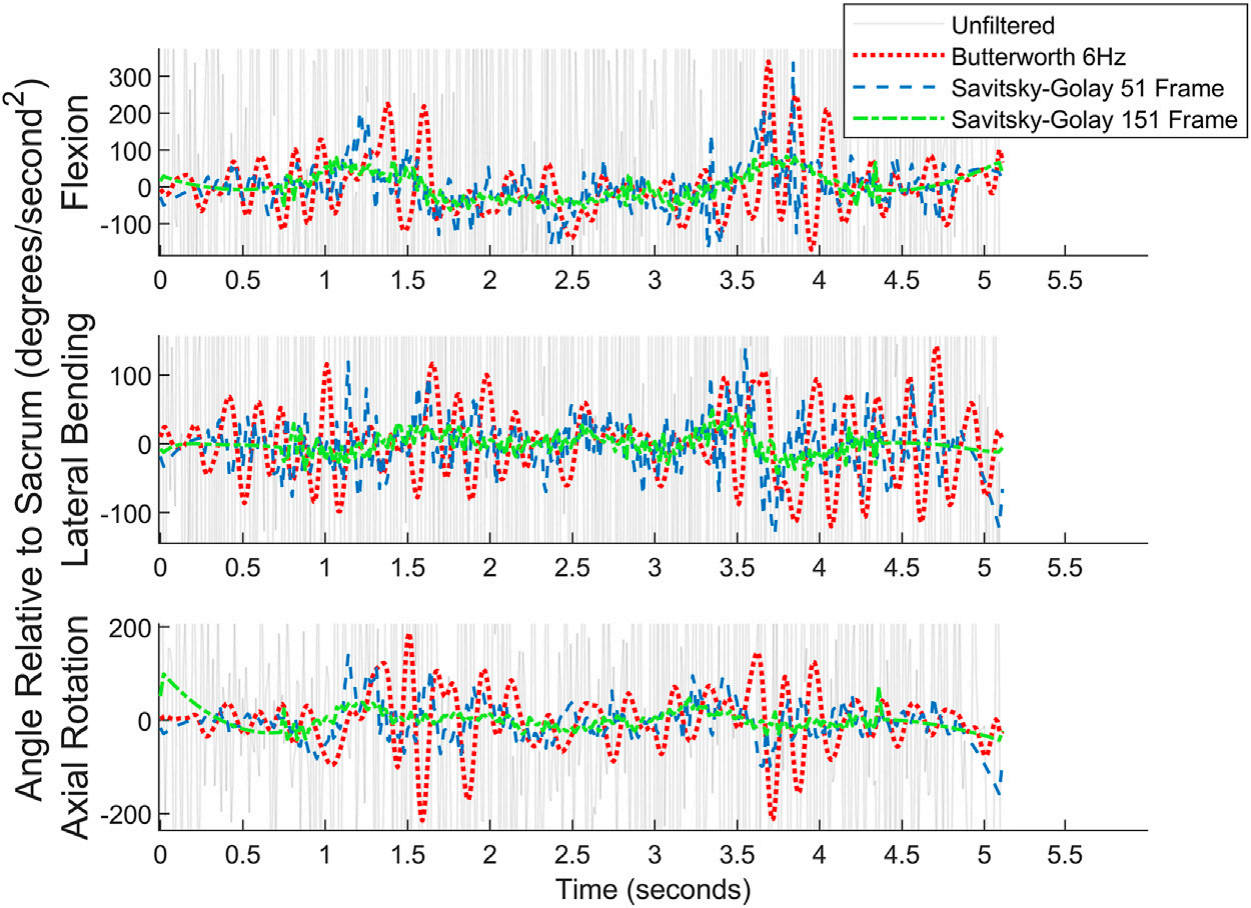
Angular acceleration data for picking up a box on the left for one subject. Raw data are overlaid with the filtered data. For readability, the *y*-axis cuts off much of the unfiltered data.

Both a 6 Hz low-pass Butterworth filter and a smaller sized,51-frame, Savitsky-Golay filter were considered before settling on the final Savitsky-Golay filter used in the present work (see Figures 11, 12). As can be observed in the figures, the process of smoothing the coordinates affects the velocity and acceleration calculations. Data obtained using the final choice of filter compared favorably with peak velocities and accelerations found in previously published studies (Ma et al., 2009; Tsang et al., 2017).

### 4.6 Limitations

In addition to skin artifacts, inherent shortcomings with the motion capture setup led to incomplete data for some trials. Diagnostic movements exhibiting large amounts of lumbar extension were difficult to capture, as the immovable cameras were all placed above the subjects. As subjects extended their spine, their body obscured the markers and caused them to drop out. This difficulty with marker dropout in extension can be seen most clearly in ExtR in Figure 8 as any errors in the marker tracking are amplified in taking the derivative to obtain velocity and acceleration. To compensate, some subjects were told to stop extension movements before end ROM to preserve useable data. Another concern inherent in this work is the reference point used in measuring the segmental motion of the spine. Initial marker placements had included markers on the S1 vertebra, but proximity to the S2 and L5 markers caused marker merging in the data processing of pilot tests. As a result, the two most caudal rows of markers were placed in line with the S2 and L5 vertebrae, and all angle measurements were made relative to the plane created by these marker rows. Using this method does not allow for direct measurement of the S1/L5 segment and leaves out important data about segmental ROM, as well as pelvic motion.

Other limitations arise from using indirect measurement methods to examine spinal kinematics. All skin-mounted biomechanical measurement techniques for measuring joint motion (e.g., accelerometers, inertial measurement units, magnetic motion tracking systems, optical marker based motion tracking systems) are indirect measures of kinematics. However, in addition to the concerns that arise when tracking systems of few joints, such as a knee or hip, there are additional challenges when extrapolating skin-mounted measurements to obtain the kinematics of spinal segments. Each spinal segment is a 3-joint complex (intervertebral disc +2 facet joints), and the region evaluated in the present work spans 6 of these 3-joint complexes. Due to this challenge, the typical soft-tissue artifacts and concerns regarding accuracy of marker placement can be amplified. Thus, the kinematic results from the present work must be framed in the context of these limitations.

Despite recent advances, motion capture systems require a large footprint, are expensive, and collecting and analyzing data is both time-consuming and requires skilled labor. Moreover, clinical implementation of a technique such as that discussed in the present work requires further evaluation of accuracy and validity, as well as inter- and intra-operator reliability studies performed with “true” reference points such as radiographs at the endpoints of each movement. Additional work quantifying STAs in these movement patterns should be performed such as examining STAs in similar multiplanar movements or an attempt to subgroup STAs for individuals based on soft tissue thickness or other relevant metrics. These challenges are non-trivial, but may be resolved as new technologies, including recent advances in wearable technologies, become more readily available. The goal of this work was to provide baseline data reporting dynamic ROM data for healthy subjects that could be used for comparison data in the future. However, when looking at individual trials, people do not perform the diagnostic movements as smoothly as the aggregate data indicate. Future work could examine typical ranges of deviation from a smooth curve and could try to classify any sub-phenotypes of movement. Further work would also benefit from examining larger cohorts with subsets in age, gender, and BMI. Data files from this study are available for analysis by other researchers (see Section 10—Data Availability Statement).

## 5 Conclusion

To our knowledge, segmental kinematic data of the lower spine for non-flexion-extension movements have not previously been reported as measured by skin-mounted optical motion tracking. This work outlines a practical methodology with a simple marker set for gathering these data in a non-invasive manner and measured range of motion values similar to those in previously published work, strengthening confidence in the accuracy of the measurements. Values for segmental end ROM varied between 24% greater to 101% smaller from previously published data, with values in the sagittal plane aligning most closely. Peak and average angular velocity values of the present work bracket the average lumbar angular velocity values reported in previously published data, and the differences are as expected due to methodological differences. This work provides a baseline of lower spine segmental kinematic data for a cohort of asymptomatic subjects in a series of single-plane and combined plane diagnostic movements that put the lower spine through its full range of motion. Future applications of these data may include comparison with symptomatic chronic or acute low back pain patients as a window into identifying diagnostic or treatment phenotypes, or as a tool for tracking success of a treatment in returning a patient to a healthy motion profile.

## Data Availability

The datasets generated and analyzed for this study can be found in the openICPSR repository (https://www.openicpsr.org/openicpsr/project/189461/version/V1/view).
